# Developmental Assessment in Children at Higher Likelihood for Developmental Delays - Comparison of Parent Report and Direct Assessment

**DOI:** 10.1007/s10803-024-06420-4

**Published:** 2024-06-14

**Authors:** Kevin G. Stephenson, Kerrigan C. Vargo, Nicole M. Cacciato, Charles M. Albright, Elizabeth M. Kryszak

**Affiliations:** 1 Child Development Center, Department of Pediatrics, Nationwide Children’s Hospital, 700 Children’s Dr, Columbus, OH 43205 USA; 2 Departments of Pediatrics and Psychology, The Ohio State University, Columbus, OH USA

**Keywords:** Parent Report, Direct Assessment, Developmental Delay

## Abstract

**Purpose:**

Accurate assessment of cognitive development of young children is a vital component of developmental evaluations. Direct assessment of developmental skills is not always feasible, but there is limited information on the agreement between direct assessment and caregiver-reported cognitive skills. There is limited information regarding the accuracy of the parent-reported Developmental Profile 4 (DP-4) in comparison to the widely-used developmental measure, the Bayley Scales of Infant and Toddler Development, Fourth Edition (Bayley-4). The purpose of the current study was to evaluate whether a standardized parent interview can effectively identify children at risk for cognitive developmental delays.

**Methods:**

We compared the agreement between the Bayley-4 Cognitive and the Developmental Profile 4 (DP-4) in young children being evaluated in-person for early developmental delays. 182 children (134 with an autism diagnosis), ages 6–42 months, completed both assessments.

**Results:**

Results showed that Bayley-4 Cognitive scores had a moderately strong correlation with DP4-Cognitive scores (*r* = 0.70, *p* < 0.001). A cutoff of 70 or 69 on the DP-4 Cognitive was determined as ideal for identifying developmental delay based on diagnosis of global developmental delay or the Bayley-4 Cognitive.

**Conclusions:**

Our analyses revealed good agreement between DP-4 and Bayley-4 Cognitive scores, even after controlling for confounding variables such as degree of ASD characteristics, age, and sex. These results suggest that caregiver-report measures can be a valid and useful tool in the assessment of young children, particularly when direct developmental assessment is not feasible.

## Introduction

Identifying early developmental delays has been recognized by medical organizations such as the American Academy of Pediatrics (AAP) as an essential way to promote well-being of all children (Lipkin et al., 2020). The AAP recommends routine screening for developmental conditions at 9-, 18- and 30-month well-child visits (Lipkin et al., 2020) in addition to screening specifically for autism spectrum disorder (ASD) at 18- and 24-month visits (Hyman et al., 2020). After children are identified as having higher risk for developmental concern, a comprehensive evaluation should follow. Important developmental domains for young children include cognition, language and communication, physical development (including fine and gross motor skills), social/emotional functioning, and adaptive skills (Alpern, 2020; Bayley & Aylward, 2019).

Developmental testing can provide awareness of which children need early intervention services. Developmental testing can also be used to predict cognitive functioning in later childhood. In a large study with a representative US sample, scores on the Mullen Scale of Early Learning (Mullen, 1995) at 2 years of age were moderately correlated with Stanford-Binet 5th Edition Abbreviated Battery IQ (Roid, 2003) scores at age 6 (*r* = 0.46). Notably, Mullen scores at one month had a substantially lower relationship with future IQ (*r* = 0.17). Similar results were found in a German sample of typically-developing children with correlations between FSIQ at 4 years and third edition of the Bayley Scales of Infant Development at 18 months (*r* = 0.43) and 26 months (*r* = 0.50) (Klein-Radukic & Zmyj, 2023). The relationship between developmental and future cognitive functioning is higher for very preterm or low birth weight children, based on the results of a meta-analysis (aggregated *r* = 0.61) (Luttikhuizen dos Santos et al., 2013).

Early detection of developmental delays leads to earlier access to appropriate intervention and, in turn, better long-term outcomes for the health of the individual (Orinstein et al., 2014). Similarly, studies have shown that early intervention and level of cognitive functioning are the most significant variables predicting outcomes for children with developmental delays and ASD (Dawson, 2008; Rogers et al., 2012). However, access to early evidence-based interventions, especially intensive therapies based on applied behavior analysis, typically requires a detailed assessment of development and formal diagnosis obtained through a specialized clinic (Alfuraydan et al., 2020). Unfortunately, there are often extensive delays between first concerns related to developmental delays and detailed assessments. These long periods of waiting have been explained by many factors, including a shortage of appropriately trained healthcare professionals and lengthy evaluations composed of several appointments (Crane et al., 2016; Thomas et al., 2007). There are also disparities in wait times and access to care for individuals from minority backgrounds (Aylward et al., 2021; Liu et al., 2023). One promising solution to the access-to-care issue is the use of telehealth. Using telehealth can decrease the wait time for referrals from primary care to connection with specialist care (Pfeil et al., 2023) and can also significantly reduce no-show rates in medical care, particularly among Black individuals (Sumarsono et al., 2023). However, the data on the appropriateness of telehealth-based methods for developmental assessments is needed to ensure that these alternative service delivery models are equivalent to existing in-person models in quality.

Although still in the early stages of empirical support, initial studies provide encouraging results for the validity of telehealth approaches for evaluating and diagnosing developmental conditions. Valentine et al. (2021) completed a systematic review of telehealth services for assessment, monitoring, and treatment of individuals with neurodevelopmental disorders. They found preliminary evidence for the accuracy of telehealth evaluations in diagnosing ASD, with one study showing an increased likelihood of families attending appointments via telehealth, strong provider and family satisfaction, as well as time to diagnosis being reduced by 11–12 months (Stainbrook et al., 2019). A second systematic review by Liu and Ma (2022) summarizes evidence for the screening and diagnostic validity of several telehealth tools. In addition to having accurate diagnostic assessments, such as those used in ASD, having valid ways of evaluating developmental skills in telehealth settings is also important.

Two primary sources of information used for assessing development in pediatric populations are parent report and direct assessment (Miller et al., 2017; Nordahl-Hansen et al., 2014). As the length of waiting time for families referred to complete clinical evaluations continues to grow, parent-report measures may serve as a time- and cost-efficient method for characterizing development for children who require immediate access to intervention. Parent-report measures are an attractive option in healthcare as they are quick, easy to use, and more cost-effective compared to direct assessments (Nordahl-Hansen et al., 2014). Additionally, parent report data can bring forth historical and functional perspectives that are not naturally accessible in a clinical testing environment (Ebert, 2017). Finally, parent-report assessment allows for better access for families in remote locations, as well as during unprecedented events, which has been highlighted through the COVID-19 pandemic, and lends itself well to use in telehealth contexts.

Despite the benefits of using parent report measures for assessing developmental delays, there may be some limitations. Some providers may consider parent report to be subjective as it reflects caregiver perception of their child’s functioning, however data suggests that parent report of language ability can be a valid and efficient tool (Sachse & Suchodoletz, 2008). Several other studies have found strong agreement between parent report and direct assessment for language and fine motor ability (Bennetts et al., 2016; James et al., 2023; Miller et al., 2017; Nordahl-Hansen et al., 2014; Sachse & Suchodoletz, 2008). There is evidence that parent report might be most accurate for children at the extreme ends of language ability (i.e., very low or very high; Bennetts et al., 2016). There is mixed evidence for diagnostic differences in the degree of agreement of direct assessment and parents report, with a recent study using a large dataset showing possible nuanced differences among children with ASD, autistic features, or developmental delay (James et al., 2023). Specifically, when matching diagnostic subgroups on sex assigned at birth, age, and nonverbal IQ, James et al. (2023) found that fine motor skills were rated lower by caregivers, compared to direct assessment, in children with ASD, autistic features, and developmental delays and receptive language skills were rated lower, compared to direct assessment, in children with ASD and autistic features. Effects sizes of these differences were small to moderate.

On the other hand, there is less research on the agreement between parent report and direct assessment of cognitive abilities in children. A recent study found evidence of a strong ability of parents to recall specific IQ scores their children received in previous testing (∼ 75% agreement; Lee et al., 2023). Estimating cognitive level relative to age or grade (e.g., above age or grade level, at age or grade level, slightly below age or grade level, and significantly below age or grade level in most abilities) resulted in 65% agreement with standardized testing (Lee et al., 2023). However, parents’ judgements of their children’s ability in this study may have been informed by previous assessments. In another study (Chandler et al., 2016), researchers asked parents to estimate the functional age or developmental age of their children with either ASD or ADHD + ID. This was converted to a developmental quotient and compared to standardized IQ testing. The majority (74%) of parents in the ADHD + ID group were able to estimate their child’s intellectual functioning within one standard deviation (i.e., 15 IQ points) whereas only 58% of parents in the ASD group estimated within one standard deviation. However, the autistic parents’ estimate might have been more based on adaptive functioning rather than cognitive functioning.

Using structured and standardized parent-report measures may lead to a higher agreement. A study of two-year-olds (Saudino et al., 1998) found a parent-report measure of non-verbal cognitive abilities created by the researchers correlated at *r* = 0.49 with a direct measure of early cognitive abilities (Mental Scale of the Bayley Scales of Infant Development-II). However, our review of the current peer-reviewed literature did not result in finding other studies making such a comparison between parent-report and direct assessment of child cognitive abilities. So, while there is some recent emerging research on the agreement of information from parents and direct assessment of early cognitive ability in children, there is a current lack of studies evaluating *standardized* parent ratings of cognitive ability and the relationship with direct measures.

### Aims of the Current Study

The primary aim of the current study was to evaluate whether a standardized parent interview can accurately identify children at risk for cognitive developmental delays. We specifically investigated retrospective clinical data from in-person evaluations which included the parent-reported Developmental Profile 4 (DP-4; Alpern, 2020) in comparison to the widely used direct assessment developmental measure, the Bayley Scales of Infant and Toddler Development, Fourth Edition (Bayley-4; Bayley & Aylward, 2019). Although the present study did not involve telehealth testing procedures, our goal was to find information on the validity of the DP-4, which can be easily administered either in person or through telehealth. The main hypotheses for the study were that the DP-4 would significantly correlate with the Bayley-4 Cognitive, show strong diagnostic accuracy compared to both a clinical diagnosis of global developmental delay (GDD) as well as a cutoff for significant developmental delay on the Bayley-4 Cognitive (Standard Score ≤ 70), and display acceptable sensitivity and specificity (i.e., sensitivity + specificity ≥ 1.5; Power et al., 2013).

## Method

### Measures

#### Bayley Scales of Infant Development, Fourth Edition (Bayley-4; Bayley & Aylward, 2019)

The Bayley-4 is a norm-referenced developmental assessment for young children. The Bayley-4 contains five scales: Cognitive, Language, Motor, Social-Emotional, and Adaptive Behavior. The evaluations for this study specifically included clinically administered Bayley-4 Cognitive scores. The Cognitive scale measures early cognitive processing skills, including item exploration and manipulation, sensorimotor development, memory, concept formation, and object relatedness. As reported in the manual (Bayley & Aylward, 2019), the Bayley-4 was highly correlated with the previous version of the Bayley (Bayley-III; corrected *r* = 0.70) and with FSIQ (*r* = 0.79) on the Wechsler Preschool and Primary Scale of Intelligence, 4th Edition (WPPSI-IV). The Bayley-4 scales also have high classification accuracy (82%) for identifying children with developmental delays. As reported in the Bayley-4 manual, test-retest reliability for the Cognitive scale across different ages ranges from *r* = 0.80 – 0.83 and internal consistency is high (average *r*_xx_ = 0.95).

#### Developmental Profile, Fourth Edition (DP-4; Alpern, 2020)

The DP-4 is a norm-referenced assessment that provide standardized information about development functioning across five domains (Physical [37 items], Adaptive Behavior [41 items], Social-emotional [36 items], Cognitive [42 items], and Communication [34 items]) for individuals from birth through 21 years. There is also a General Development Score which is a composite of general developmental ability across the five domains. Ratings for items are based on a dichotomous format (i.e., Yes/No) of a particular skill being present. Higher scores indicate better developmental skills. The standardization sample was based on 2,259 cases with a demographic breakdown similar to the 2019 U.S. Census. There are four forms including Parent/Caregiver Interview, Parent/Caregiver Checklist, Teacher Checklist, and Clinician Rating. For the current study, only the Parent/Caregiver Interview Form was used. Internal consistency for the interview form is high (*r* = 0.80 – 0.98). Test-retest reliability (average of two weeks) of the DP-4 is generally fair to good (*r* = 0.65 – 0.84). There is also evidence of validity of the DP-4 supported by exploratory common factor analysis, correlations with the previously published version (5 domains: *r*s = 0.80 − 0.89; General Development: *r* = 0.93), and with another developmental measure (i.e., Developmental Assessment of Young Children, Second Edition; DAYC-2; domains: *r*s = 0.49 − 0.67; General Development: *r* = 0.64).

### Procedure

#### Participants

Participant characteristics are found in Table 1. Participants in the study included 167 children (60 female, 35.9%), between 6 and 42 months old, referred for an in-person developmental evaluation between September 2021 through May 2023 at a large pediatric hospital in the Midwestern United States. The sample was racially diverse (56.3% White, 24.6% Black/African American, 10.2% Bi-racial/Multi-racial, 6.6% Asian, 2.4% Unknown). Most participants received a diagnosis of autism spectrum disorder (*n* = 122, 73.1%). A small number of children were evaluated after having non-accidental head trauma (*n* = 15, 9%). Level of autistic traits, as measured by the Childhood Autism Rating Scale, Second Edition (CARS-2; Schopler et al., 2010), was available for 80% (*n* = 133) of the sample. The CARS-2 is a clinician-rated measure of autistic traits based on both direct observation as well as information from caregivers. Higher scores reflect greater degree of autistic symptoms. Data were obtained through retrospective chart review of patients referred for an evaluation due to developmental delay and were administered both the DP-4 and Bayley-4 in person. The hospital’s Institutional Review Board (IRB) approved this retrospective study.

**Table 1:** Participant characteristics

	Non-ASD (*n* = 45)	ASD (*n* = 122)	Overall (*N* = 167)
Biological Sex			
Female	19 (42.2%)	41 (33.6%)	60 (35.9%)
Male	26 (57.8%)	81 (66.4%)	107 (64.1%)
Age (in months)			
Mean (SD)	24.4 (11.1)	32.7 (6.11)	30.4 (8.54)
Median [Min, Max]	26.0 [6.00, 42.0]	33.0 [19.0, 42.0]	32.0 [6.00, 42.0]
Race			
Asian	3 (6.7%)	8 (6.6%)	11 (6.6%)
Bi-racial/Multi-racial	4 (8.9%)	13 (10.7%)	17 (10.2%)
Black	9 (20.0%)	32 (26.2%)	41 (24.6%)
White	29 (64.4%)	65 (53.3%)	94 (56.3%)
Missing	0 (0%)	4 (3.3%)	4 (2.4%)
GDD Diagnosis			
No	30 (66.7%)	20 (16.4%)	50 (29.9%)
Yes	15 (33.3%)	102 (83.6%)	117 (70.1%)
Language Disorder Diagnosis			
No	40 (88.9%)	115 (94.3%)	155 (92.8%)
Yes	5 (11.1%)	7 (5.7%)	12 (7.2%)
Head Trauma			
No	30 (66.7%)	122 (100%)	152 (91.0%)
Yes	15 (33.3%)	0 (0%)	15 (9.0%)
Bayley-4 Cognitive Standard score			
Mean (SD)	79.0 (16.1)	63.5 (11.9)	67.7 (14.8)
Median [Min, Max]	80.0 [55.0, 115]	55.0 [55.0, 100]	65.0 [55.0, 115]
DP-4 Cognitive Standard Score			
Mean (SD)	84.7 (16.5)	62.8 (13.6)	68.7 (17.4)
Median [Min, Max]	91.0 [40.0, 110]	61.0 [40.0, 97.0]	64.0 [40.0, 110]
CARS-2 Raw Score			
Mean (SD)	26.0 (8.02)	36.9 (4.38)	35.4 (6.25)
Median [Min, Max]	25.5 [17.0, 50.0]	37.0 [25.5, 48.5]	36.0 [17.0, 50.0]
Missing	27 (60.0%)	7 (5.7%)	34 (20.4%)

Note. GDD = Global Developmental Delay; DP-4 = Developmental Profile 4; CARS-2 = Childhood Autism Rating Scale, 2nd Edition

#### Clinical Evaluation Procedures

All children were evaluated by English-speaking clinicians through routine, standard of care developmental evaluations. Evaluations were completed by psychology providers who consisted of clinical psychologists with extensive experience in neurodevelopmental assessment or pre- or post-doctoral psychology trainees supervised by clinical psychologists and typically consisted of a single day of evaluation. Trained psychometricians (bachelor’s or master’s level technicians) under the supervision of clinical psychologists assisted in administration of the Bayley-4. All DP-4 interviews were completed by psychology providers. Interpreters were used for the DP-4 interview with caregivers who did not speak English (3 Somali, 3 Nepali, 2 Spanish, 1 French, 1 Hindi, and 1 Urdu). Ratings on the CARS-2 were based on both direct in-person observations of and interactions with the child as well as caregiver report from a clinical interview. Final clinical diagnoses were based on expert clinical judgement integrating data from standardized assessments, information from a clinical interview, and available collateral information (e.g., review of medical record).

#### Statistical Analysis

We used Pearson correlations to investigate bivariate relationships between DP-4 subscales, Bayley-4 Cognitive, and CARS-2 scores. Using multiple regression we predicted Bayley-4 Cognitive scores using DP-4 subscale scores. CARS-2 scores, age, and biological sex were also included as covariates. We then ran a regression model with all DP-4 subscale scores as predictors of Bayley-4 Cognitive scores to find the best overall predictor. Raw scores were used for correlation and regression analyses due to floor effects for standardized scores in our sample which would have resulted in a restriction of range in the analyses. The accuracy of the DP-4 Cognitive scale in identifying significant developmental delay as defined by clinical diagnosis of GDD. However, due to the retrospective nature of the data, we did not have DP-4 ratings that were independent of the final diagnostic decision. We also evaluated the accuracy of the DP-4 as measured by the Bayley-4 Cognitive scale (SS ≤ 70) as an independent measure given that the information from the DP-4 did not influence the Bayley-4 administration. We completed a receiver operating characteristic (ROC) analysis including calculating the area under the ROC curve (AUC). We also calculated sensitivity, specificity, positive predictive value (PPV), and negative predictive value (NPV) of the DP-4 Cognitive scale (Standard Score). Data were analyzed using R v.4.3.0. Accuracy analyses were completed using the *pROC* package (Robin et al., 2011, 2023).

## Results

### Correlation and Regression Analyses

**Fig. 1:**
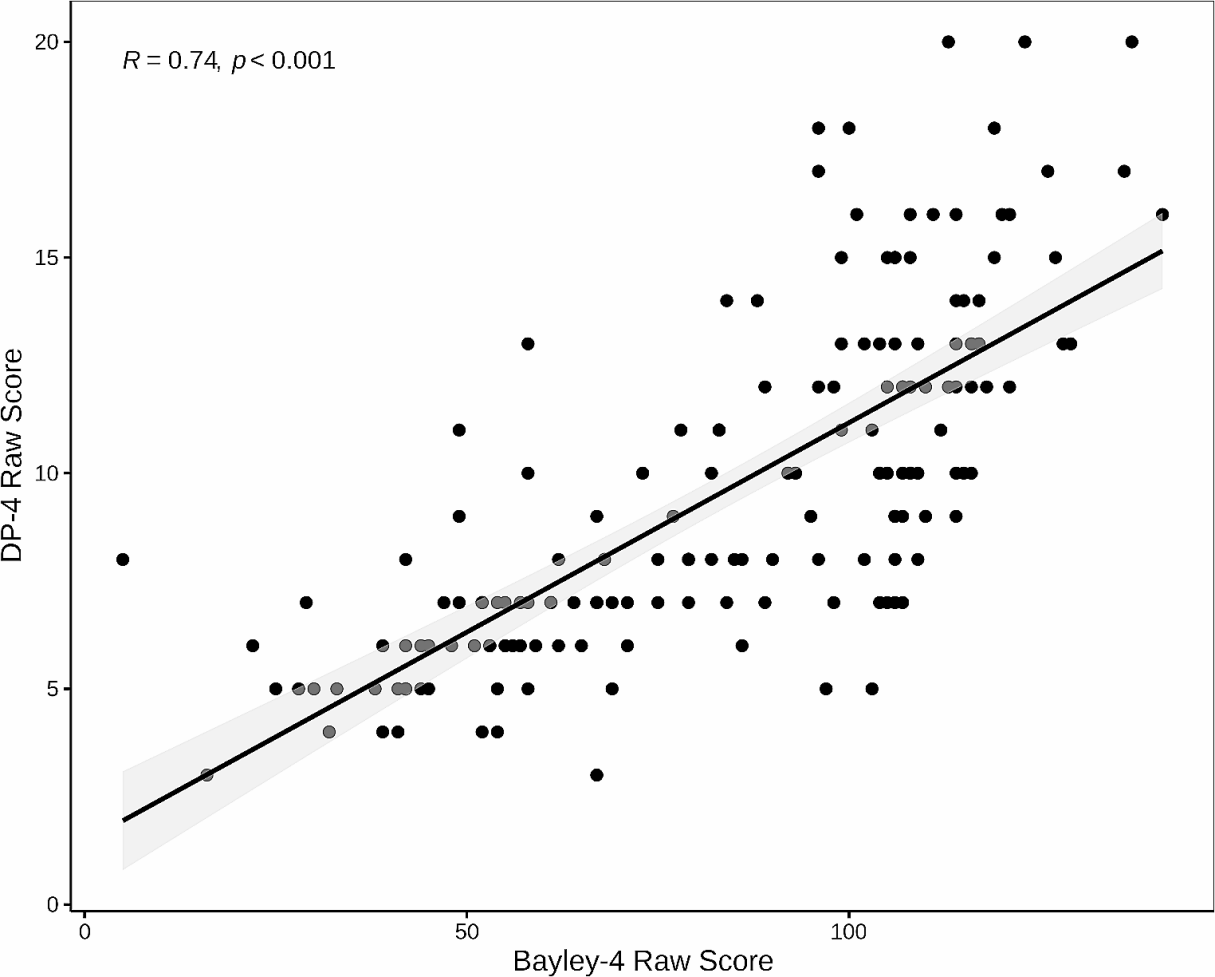
Scatter plot of DP-4 and Bayley-4 raw cognitive scores

Bayley-4 Cognitive scores were significantly and positively correlated with all DP-4 subscales (see Table 2). Among the DP-4 subscales, the Cognitive score had the strongest correlation with the Bayley-4 Cognitive score (*r* = 0.70, 95% CI [0.60, 0.78]; see Fig. 1). This relationship continued to be significant after controlling for CARS-2 scores, age, and sex (β = 0.42, 95% CI [0.28, 0.56]). Age (β = 0.22, 95% CI [0.10, 0.33]) and CARS-2 scores (β = − 0.37, 95% CI [-0.50, − 0.24]) were also significant predictors in the multiple regression model. When all DP-4 subscales were included as predictors of Bayley-4 scores (i.e., accounting for shared variance between the scales), the DP-4 Cognitive scale remained the best overall predictor among the DP-4 subscores (β = 0.32, 95% CI [0.10, 0.53]). The DP-4 Physical score also predicted unique variance, but to a lesser degree (β = 0.17, 95% CI [0.01, 0.33]).

**Table 2:** Correlations for study variables

Variable	1	2	3	4	5	6
1. Bayley-4 Cognitive	–					
2. DP4-Cognitive	0.70	–				
3. DP4-Physical	0.56	0.56	–			
4. DP4-Social	0.42	0.64	0.38	–		
5. DP4-Communication	0.62	0.82	0.43	0.69	–	
6. DP4-Adaptive	0.52	0.50	0.68	0.40	0.46	–
7. CARS-2	− 0.65	− 0.56	− 0.33	− 0.37	− 0.56	− 0.39

Note. Correlations between raw scores. DP4 = Developmental Profile 4; CARS-2 = Childhood Autism Rating Scale, Second Edition. All correlations were significant at level *p* < 0.001

### Classification Accuracy (ROC Analysis)

**Fig. 2:**
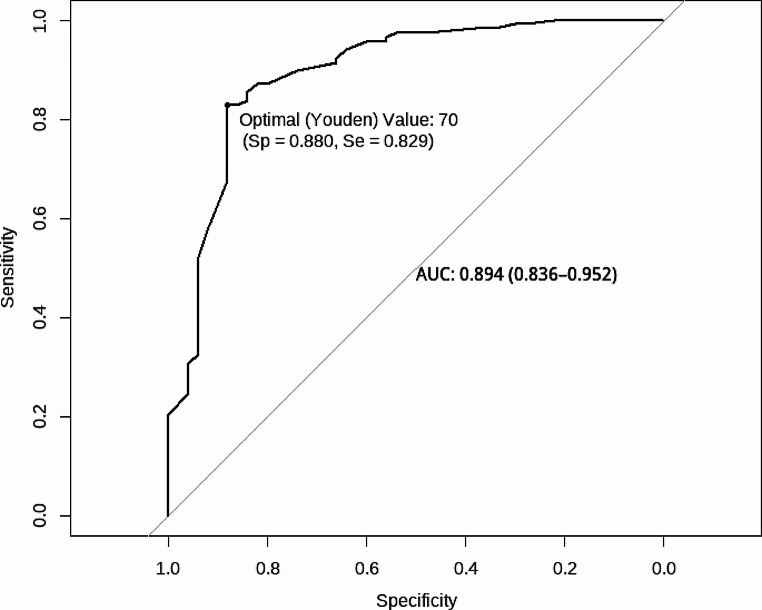
DP-4 cognitive receiver operating curve (ROC) plot for global developmental delay diagnosis

**Fig. 3:**
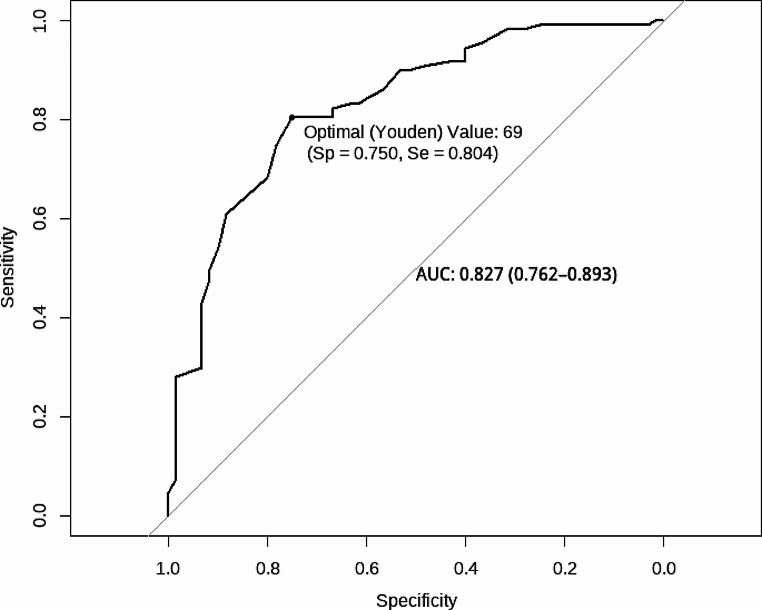
DP-4 cognitive receiver operating curve (ROC) plot for bayley-4 cognitive score

Sensitivity/specificity analyses are summarized in Table 3 (for GDD diagnosis) and Table 4 (for Bayley-4 Cognitive score). A similar optimal cut-off (based on Youden criteria) on the DP-4 Cognitive emerged for predicting both GDD diagnosis (SS = 70) and the Bayley-4 Cognitive score ≤ 70 (SS = 69). For predicting GDD diagnosis, the DP-4 had an AUC of 0.894 (95% CI [0.836, 0.952]) with a specificity of 0.88 and a sensitivity of 0.83 (see Fig. 2). The AUC for predicting Bayley-4 Cognitive ≤ 70 was 0.827 (95% CI [0.762, 0.893]) with a specificity of 0.75 and sensitivity of 0.80 (see Fig. 3). We compared the ROC curves of those with ASD to those without ASD using DeLong’s test and there were no significant differences for the GDD outcome [*D*(147.12) = -0.96, *p* = 0.34] or the Bayley-4 Cognitive outcome [*D*(80.31) = 0.41, *p* = 0.69].

**Table 3:** Accuracy results – based on clinical diagnosis of global developmental delay

DP-4 Cognitive Standard Score	TP	FP	TN	FN	Se	Sp	NPV	PPV
90	114	26	24	3	0.97	0.48	0.89	0.81
89	114	23	27	3	0.97	0.54	0.90	0.83
88	113	22	28	4	0.97	0.56	0.88	0.84
86	112	22	28	5	0.96	0.56	0.85	0.84
84	112	20	30	5	0.96	0.60	0.86	0.85
83	110	18	32	7	0.94	0.64	0.82	0.86
82	108	17	33	9	0.92	0.66	0.79	0.86
81	107	17	33	10	0.91	0.66	0.77	0.86
80	105	13	37	12	0.90	0.74	0.76	0.89
78	104	12	38	13	0.89	0.76	0.75	0.90
77	103	11	39	14	0.88	0.78	0.74	0.90
76	102	10	40	15	0.87	0.80	0.73	0.91
75	102	9	41	15	0.87	0.82	0.73	0.92
74	100	8	42	17	0.85	0.84	0.71	0.93
73	99	8	42	18	0.85	0.84	0.70	0.93
72	98	8	42	19	0.84	0.84	0.69	0.92
71	97	7	43	20	0.83	0.86	0.68	0.93
70	97	6	44	20	0.83	0.88	0.69	0.94
69	95	6	44	22	0.81	0.88	0.67	0.94
67	87	6	44	30	0.74	0.88	0.59	0.94
65	79	6	44	38	0.68	0.88	0.54	0.93
63	68	4	46	49	0.58	0.92	0.48	0.94
61	61	3	47	56	0.52	0.94	0.46	0.95
59	55	3	47	62	0.47	0.94	0.43	0.95
58	53	3	47	64	0.45	0.94	0.42	0.95
57	47	3	47	70	0.40	0.94	0.40	0.94
55	43	3	47	74	0.37	0.94	0.39	0.93
54	38	3	47	79	0.32	0.94	0.37	0.93
53	36	2	48	81	0.31	0.96	0.37	0.95
52	34	2	48	83	0.29	0.96	0.37	0.94
51	29	2	48	88	0.25	0.96	0.35	0.94
50	24	0	50	93	0.21	1.00	0.35	1.00

Note. DP4 = Developmental Profile 4; TP = true positives; FP = false positives; TN = true negatives; FN = false negatives; Se = sensitivity; Sp = specificity; NPV = negative predictive value; PPV = positive predictive value

**Table 4:** Accuracy results – based on score of ≤ 70 on the bayley-4 cognitive scale

DP-4 Cognitive Standard Score	TP	FP	TN	FN	Se	Sp	NPV	PPV
90	102	38	22	5	0.95	0.37	0.81	0.73
89	101	36	24	6	0.94	0.40	0.80	0.74
88	99	36	24	8	0.93	0.40	0.75	0.73
86	98	36	24	9	0.92	0.40	0.73	0.73
84	98	34	26	9	0.92	0.43	0.74	0.74
83	97	31	29	10	0.91	0.48	0.74	0.76
82	96	29	31	11	0.90	0.52	0.74	0.77
81	96	28	32	11	0.90	0.53	0.74	0.77
80	92	26	34	15	0.86	0.57	0.69	0.78
78	91	25	35	16	0.85	0.58	0.69	0.78
77	90	24	36	17	0.84	0.60	0.68	0.79
76	89	23	37	18	0.83	0.62	0.67	0.79
75	89	22	38	18	0.83	0.63	0.68	0.80
74	88	20	40	19	0.82	0.67	0.68	0.81
73	87	20	40	20	0.81	0.67	0.67	0.81
72	86	20	40	21	0.80	0.67	0.66	0.81
71	86	18	42	21	0.80	0.70	0.67	0.83
70	86	17	43	21	0.80	0.72	0.67	0.83
69	86	15	45	21	0.80	0.75	0.68	0.85
67	80	13	47	27	0.75	0.78	0.64	0.86
65	73	12	48	34	0.68	0.80	0.59	0.86
63	65	7	53	42	0.61	0.88	0.56	0.90
61	58	6	54	49	0.54	0.90	0.52	0.91
59	53	5	55	54	0.50	0.92	0.50	0.91
58	51	5	55	56	0.48	0.92	0.50	0.91
57	46	4	56	61	0.43	0.93	0.48	0.92
55	42	4	56	65	0.39	0.93	0.46	0.91
54	37	4	56	70	0.35	0.93	0.44	0.90
53	34	4	56	73	0.32	0.93	0.43	0.89
52	32	4	56	75	0.30	0.93	0.43	0.89
51	30	1	59	77	0.28	0.98	0.43	0.97
50	23	1	59	84	0.21	0.98	0.41	0.96

Note. DP4 = Developmental Profile 4; TP = true positives; FP = false positives; TN = true negatives; FN = false negatives; Se = sensitivity; Sp = specificity; NPV = negative predictive value; PPV = positive predictive value

## Discussion

The current study aimed to evaluate the consistency between parent report and direct assessment of child development. Overall, our results suggest that parent report of child cognitive ability is significantly related to and reliably predictive of a clinical diagnosis of GDD and performance on a direct assessment measure of cognitive development. When investigating an optimal cut-off point on the DP-4, a Scaled Score of 70 or 69 optimized sensitivity while maintaining an acceptable level of specificity when using these measures in a clinical context with a high base rate of developmental delay. These values align with typical clinical cutoffs such as “significantly delayed”, as commonly defined in clinical contexts by 2 standard deviations below the mean.

In our sample, we found that age of the child and level of autistic traits were significant predictors of Bayley-4 Cognitive scores, above and beyond DP-4 Cognitive scores. Older children generally had higher cognitive functioning, which is expected given our use of raw scores. The negative relationship between autistic traits and developmental abilities has been seen in other work (e.g., Shan et al., 2022) and highlights possible patterns of global delays in children with more profound autism traits.

The DP-4 Cognitive scale had an acceptable level of predictive accuracy in identifying children with developmental delays. However, after accounting for overlap (shared variance) between DP-4 subscales, the Physical scale also predicted unique variance in Bayley-4 Cognitive scores in a multiple regression analysis. Although we did not anticipate this finding, previous research has shown a relationship between motor skills and cognitive functioning in toddlers (Martzog et al., 2019; Veldman et al., 2019). Further research should explore this finding to better understand the relationship between motor functioning and cognitive skills, especially in children with developmental delays.

Our findings of moderately strong agreement between parent-reported and direct assessment of early cognitive development add to evidence for agreement across other developmental domains (Miller et al., 2017; Nordahl-Hansen et al., 2014; Sachse & Suchodoletz, 2008). Overall, the data from this study offer evidence that the DP-4 can be accurate in detecting developmental delays in early cognitive ability and has the potential to become an acceptable choice in routine assessment. However, there is still a need to identify specific skills as targets for intervention, which may be most feasible with in-person assessments. Despite this evidence of the utility of parent-reported developmental measures, this is not to imply that these measures can or should unilaterally replace direct assessment. Combining both direct assessment and standardized parent-report measures can increase the predictive validity of the results (Saudino et al., 1998). This follows standard guidelines from the American Psychological Association which call for multi-informant and multi-method approaches for evaluation (American Psychological Association, 2020).

### Limitations and Future Directions

Our results cannot be interpreted without identifying key limitations. While this sample included children from diverse cultural, ethnic, and language backgrounds, our sample size was not large enough to analyze potential differences within minority demographic groups. Additionally, given the young age and high prevalence of developmental delay in our sample, it is unclear how well these results will generalize to older children or those will less severe developmental delays. Further research with larger samples, particularly including a higher proportion of individuals from diverse backgrounds, is needed to confirm and expand our results. Because DP-4 scores were used among other test data in informing final diagnosis, the accuracy of DP-4 scores and diagnosis of GDD may have been artificially higher. However, we found similar results with the Bayley-4, which was independent of DP-4 ratings. While we demonstrated strong agreement between the DP-4 and Bayley-4 Cognitive scores, future research should explore agreement between the DP-4 and other cognitive measures, including IQ tests, to see if the same agreement exists at later stages of development.

### Implications

To our knowledge, this is one of few studies investigating agreement between parent report and direct assessment of child cognitive ability in a sample of children being evaluated for developmental delay. Our results suggest that parents are accurate reporters of their young child’s cognitive skills, particularly on a standardized parent interview. Additionally, degree of autism severity, age, and biological sex did not appear to impact the observed findings. While these findings demonstrate that parents are a reliable source for measuring early cognitive ability, we stress the continued need for multimodal and multi-informant evaluations when assessing development in early childhood.
